# Blood Microbiome Reveals the Impact of *Lactobacillus* on the Efficacy of Immunotherapy in Gastrointestinal Cancer

**DOI:** 10.1002/mco2.70316

**Published:** 2025-08-11

**Authors:** Ya‐Shang Zheng, Wu‐Hao Lin, Jun‐Quan Chen, Xiao‐Li Wei, Jia‐Qian Huang, Yu‐Hong Xu, Meng Yang, Qi‐Hua Zhang, Zhi‐Jun Zuo, Zhao‐Ying Yang, Pan Zhang, Nga Ki HONG, Lu‐Xuan Liu, Zhao‐Lei Zeng, Rui‐Hua Xu, Hui‐Yan Luo

**Affiliations:** ^1^ Department of Medical Oncology State Key Laboratory of Oncology in South China Collaborative Innovation Center for Cancer Medicine Guangdong Provincial Clinical Research Center for Cancer Sun Yat‐Sen University Cancer Center Guangzhou People's Republic of China; ^2^ Research Unit of Precision Diagnosis and Treatment for Gastrointestinal Cancer Chinese Academy of Medical Sciences Guangzhou People's Republic of China; ^3^ Department of Molecular Diagnostics State Key Laboratory of Oncology in South China Collaborative Innovation Center for Cancer Medicine Guangdong Provincial Clinical Research Center for Cancer Sun Yat‐Sen University Cancer Center Guangzhou People's Republic of China; ^4^ Department of Anesthesiology Sun Yat‐Sen University Cancer Center State Key Laboratory of Oncology in South China Collaborative Innovation Center for Cancer Medicine Guangzhou People's Republic of China; ^5^ Sun Yat‐Sen University Zhongshan School of Medicine Guangzhou People's Republic of China

**Keywords:** biomarker, blood microbiome, efficacy, immunotherapy, *Lactobacillus*

## Abstract

Immunotherapy has revolutionized the treatment of gastrointestinal (GI) cancers, but reliable biomarkers for predicting treatment efficacy remain limited. In this study, we explored the potential of blood microbiome and specific microbial taxa as novel biomarkers for predicting the efficacy of immunotherapy combined with chemotherapy in GI cancer patients through 16S rRNA sequencing. Our findings demonstrated that lower baseline alpha diversity and specific microbial compositions, particularly lower levels of *Lactobacillus*, were significantly associated with longer progression‐free survival (PFS) in patients receiving immunotherapy combined with chemotherapy. Furthermore, we validated the reliability of *Lactobacillus* abundance as a predictor of PFS and treatment response in another independent patient cohort. Additionally, patients with increased or stable levels of *Lactobacillus* after immunotherapy combined with chemotherapy had superior PFS. Gavage of *Lactobacillus rhamnosus (L. rhamnosus)* el evated its blood level and enhanced the efficacy of immunotherapy in mouse models. Our results suggest that *Lactobacillus* may serve as a novel biomarker for predicting the efficacy of immunotherapy combined with chemotherapy and hold the potential as a PD‐1 antibody sensitizer.

## 1 Introduction

Gastrointestinal (GI) cancers, including esophageal cancer, gastric cancer, and colorectal cancer (CRC), represent a significant global health burden. According to GLOBOCAN 2022 data, these three cancers collectively account for approximately 3.5 million new cases annually, making up about 19% of all new cancer diagnoses worldwide. In terms of mortality, these cancers are responsible for over 2.1 million deaths each year, which is nearly 25% of all cancer‐related deaths [1]. Despite the availability of chemotherapy and certain targeted therapies as standard treatments for these cancers, their effectiveness remains limited, particularly in advanced stages [2, 3]. Immunotherapy has emerged as a promising alternative. Immune checkpoint inhibitors (ICIs), particularly programmed death‐1 and its ligand (PD‐1/PD‐L1) inhibitors, have shown promising results in these malignancies. In esophageal cancer, trials reported that ICIs combined with chemotherapy yielded an objective response rate (ORR) of 47%–64% and a median overall survival (OS) of 13.2–17.0 months, particularly benefiting patients with high PD‐L1 expression [4, 5, 6]. For gastric cancer, studies demonstrated that the addition of ICIs to chemotherapy improved the OS in unresectable or metastatic GC (13.8–15.2 months) with an ORR of around 57.5%–60% [7, 8, 9]. ICIs, such as PD‐1 and CTLA‐4 inhibitors, have shown significant efficacy in the first‐line treatment of microsatellite instability‐high (MSI‐H) CRC [10]. However, their application in microsatellite‐stable (MSS) CRC has been more challenging. MSS CRC generally has a lower mutational burden and a less immunogenic tumor microenvironment, making them less responsive to ICIs. Nonetheless, there are ongoing efforts and studies exploring ways to enhance the effectiveness of ICIs in MSS CRC through combination therapies and novel therapeutic strategies [11].

Overall, while immunotherapy has transformed the treatment landscape for certain GI cancers, patient selection remains critical. The effectiveness of immunotherapy in GI cancers is influenced by several factors, including tumor biology, the tumor microenvironment, and patient‐specific characteristics. Key biomarkers used to predict the response to immunotherapy, including PD‐L1 expression, MSI status, and tumor mutational burden (TMB), have limitations. PD‐L1 expression is commonly used, but its predictive value varies across different GI cancers and can be influenced by the assay used and heterogeneous expression within tumors [12, 13]. MSI‐H status is strongly associated with response to ICIs; however, it is found in only a small proportion (approximately 4%–5%) of CRC and is even less frequent in gastric and esophageal cancers [14]. The predictive power of TMB is not consistent across all GI cancers, and there are no standard cut‐off thresholds for “high TMB” [15]. Given these challenges, researchers are exploring more comprehensive biomarkers, such as circulating tumor DNA (ctDNA), immune gene expression profiles, and novel approaches like human microbiota [16, 17]. Recent studies have highlighted the relationship between microbiota (including gut microbiota, intratumoral microbiota, and blood microbiota) and the efficacy of immunotherapy in cancer treatment. Several studies have focused on predicting immunotherapy outcomes using fecal microbiota profiles. For instance, Routy et al. (2018) found that the presence of *Akkermansia muciniphila* in the gut was associated with improved responses to PD‐1 inhibitors in lung and kidney cancers [18]. A study found that the clinical response to anti‐PD‐1/PD‐L1 immunotherapy in GI cancer patients is significantly associated with gut microbiome composition, with responders enriched in specific beneficial bacteria (e.g., *Prevotella*) [19]. Several studies highlighted the dual role of intratumoral microbiota in CRC, demonstrating its ability to either suppress or enhance antitumor immunity and immunotherapy efficacy, while proposing microbial profiling or targeted manipulation as strategies to improve therapeutic outcomes [20].

However, monitoring gut or intratumoral microbiota faces substantial hurdles in clinical practice. The gut microbiota is highly dynamic and can be influenced by diet, lifestyle, antibiotics, and other medications [21]. This variability can lead to inconsistent findings and reduced reliability in predicting immunotherapy outcomes [22]. Additionally, fecal sampling represents only a proxy for the microbial environment in the tumor microenvironment, which limits its predictive accuracy [23]. Moreover, there are challenges in monitoring intratumoral microbiota as a predictive biomarker. First, the spatial heterogeneity of tumor microbiota makes it difficult to capture a comprehensive and representative profile of the microbial community in a single biopsy [24]. Additionally, the invasive nature of obtaining tumor biopsies makes it unsuitable for long‐term monitoring [25]. In this context, the blood microbiome emerges as a promising alternative. Monitoring the blood microbiome presents a novel, noninvasive approach that provides a systemic perspective. Blood samples are minimally invasive and easily accessible, enabling regular monitoring of microbial dynamics during treatment. Additionally, the blood microbiome may provide a more comprehensive view of systemic microbial influences compared to site‐specific microbiomes like those in the gut or tumor. While gut and tumor microbiomes have traditionally been the focus of research, emerging evidence suggests that the blood microbiome may also influence systemic immune responses. A study developed circulating microbiome signatures to predict the tumor immune microenvironment and patient outcomes in non‐small cell lung cancer (NSCLC) patients receiving immunotherapy [26]. Another study found that the detection of specific bacterial DNA in blood at treatment baseline was linked to either a treatment response and clinical benefit (*Peptostreptococcae*, *Paludibaculum*, *Lewinella*) or to tumor progression (*Gemmatimonadaceae*) in NSCLC patients treated with nivolumab [27]. However, research exploring the blood microbiome's predictive role in immunotherapy outcomes for CRC patients remains notably scarce. Emerging evidence from a study revealed that elevated baseline alpha diversity of circulating microbiota was significantly correlated with improved PFS and OS in CRC patients undergoing dendritic cell/cytokine‐induced killer (DC/CIK) immunotherapy [28]. Notably, therapeutic responders demonstrated a distinct enrichment of commensal taxa within the blood microbiome, including *Bifidobacterium spp*., *Lactobacillus spp*., and *Enterococcus* spp., compared to nonresponders. Despite these preliminary findings, the utility of blood microbiome signatures in predicting responses to PD‐1/PD‐L1 inhibitors in GI cancers remains underexplored. Current literature lacks robust biomarkers or validated microbial taxa directly associated with anti‐PD‐1 efficacy, highlighting a critical knowledge gap.

Our study collected baseline and posttreatment blood samples from 134 GI cancer patients receiving either immunotherapy combined with chemotherapy or standard chemotherapy. Peripheral blood mononuclear cells (PBMCs) were analyzed using 16S rRNA sequencing and absolute quantification of blood microbiome DNA. The study aims to analyze the correlation between blood microbiome and the efficacy of immunotherapy and to identify potential strategies to enhance the effectiveness of immunotherapy in GI cancers.

## 2 Results

### 2.1 Lower Blood Microbial Alpha Diversity Is Significantly Associated With Benefit From the Combination of Immunotherapy and Chemotherapy

**FIGURE 1:**
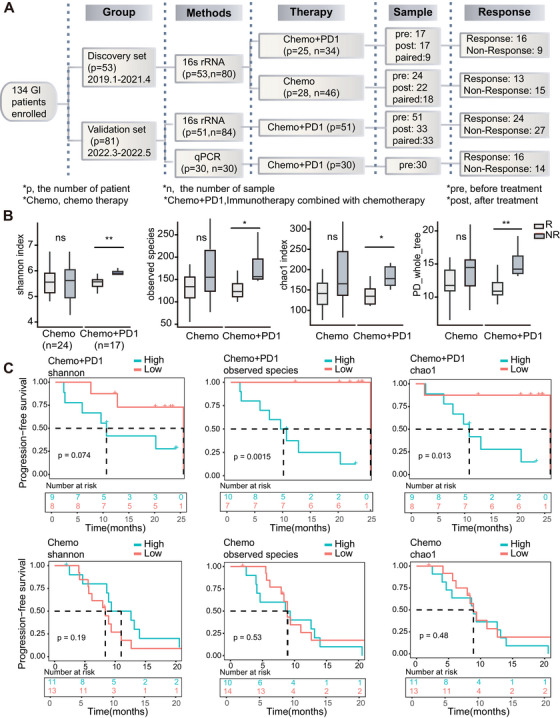
Correlation between blood microbial diversity and response to immunotherapy in gastrointestinal cancer patients. Flowchart illustrating the analysis of circulating microbiome DNA. (A) a. 16S rRNA: 80 samples from 53 patients (27 paired samples) were collected before and after treatment in the discovery set. b. 16S rRNA: 84 samples from 51 patients (33 paired samples) were collected before and after treatment in the Validation Set 1. c. qPCR analysis: 30 pretreatment samples from 30 patients were collected in the Validation Set 2. (B) Box plot comparing baseline alpha diversity indices (observed species, Shannon, Chao1, and PD_whole_tree) between responder (R) and nonresponder (NR) groups in patients treated with chemotherapy alone (Chemo, *n* = 24) and immunotherapy combined with chemotherapy (Chemo+PD1, *n* = 17). (C) Kaplan–Meier survival curves of gastrointestinal cancer patients before chemotherapy (*n* = 24) and immunotherapy combined with chemotherapy (*n* = 17), categorized by alpha diversity indices (observed species, Shannon, and Chao1). See also Figure S1.

To explore the relationship between blood microbiota and immunotherapy in patients with GI cancers and to identify potential biomarkers for predicting the efficacy of immunotherapy, our center established a cohort of patients with GI cancers. A total of 134 patients who received either chemotherapy alone (*n* = 24) or immunotherapy combined with chemotherapy (*n* = 98) were included (responders were classified as the R group and nonresponders as the NR group, Figure 1A). We stratified the patients into two cohorts based on distinct testing periods. Cohort 1, consisting of 53 patients, served as the discovery set, enabling the formulation of preliminary conclusions. Cohorts 2 and 3, comprising 81 patients, were designated as Validation Sets 1 and 2, aimed at verifying these conclusions and enhancing the accuracy of the findings. Clinical information of patients in different patient cohorts is presented in Tables 1, 2, and 3.

**TABLE 1:** Clinical characteristics of patients in the discovery set.

Clinical characteristics	Chemo (*N* = 28)	Chemo+PD1 (*N* = 25)	p.overall
Cancers			< 0.001
CRC	17 (60.7%)	0 (0.00%)	—
ESCC	8 (28.6%)	12 (48.0%)	—
GC	3 (10.7%)	13 (52.0%)	—
Gender			0.640
Female	7 (25.0%)	4 (16.0%)	—
Male	21 (75.0%)	21 (84.0%)	—
Liver metastasis			1.000
No	17 (60.7%)	16 (64.0%)	—
Yes	11 (39.3%)	9 (36.0%)	—
Number of metastatic organs			0.624
≤ 1	15 (53.6%)	16 (64.0%)	—
≥2	13 (46.4%)	9 (36.0%)	—
Age	58.9 (11.5)	58.0 (13.0)	0.792
BMI	21.9 (2.44)	21.8 (3.71)	0.970
Response:			0.314
NR	15 (53.6%)	9 (36.0%)	—
R	13 (46.4%)	16 (64.0%)	—
PFS_group:			0.725
< 6 m	9 (32.1%)	6 (24.0%)	—
> 6 m	19 (67.9%)	19 (76.0%)	—

Abbreviations: BMI, body mass index; CRC, colorectal cancer; ESCC, esophageal squamous cell carcinoma; GC, gastric cancer; NR, nonresponse group; R, response group.

**TABLE 2:** Clinical characteristics of patients in Validation Set 1.

Clinical characteristics	NR (*N* = 27)	R (*N* = 24)	p.overall
Cancers			0.019
ESCC	7 (25.9%)	15 (62.5%)	—
GC	20 (74. 1%)	9 (37.5%)	—
Gender			1.000
Female	8 (29.6%)	8 (33.3%)	—
Male	19 (70.4%)	16 (66.7%)	—
Liver metastasis			0.448
No	19 (70.4%)	20 (83.3%)	—
Yes	8 (29.6%)	4 (16.7%)	—
Number of metastatic organs:			0.456
≤ 1	13 (48. 1%)	15 (62.5%)	—
≥ 2	14 (51.9%)	9 (37.5%)	—
Age	56.6 (12.4)	59.1 (10.5)	0.433
BMI	21.8 (3.38)	21.6 (3.24)	0.797
PFS_group:			0.002
< 6#x000A0;m	17 (63.0%)	4 (16.7%)	—
> 6#x000A0;m	10 (37.0%)	20 (83.3%)	—

**TABLE 3:** Clinical characteristics of patients in Validation Set 2.

Clinical characteristics	NR (*N* = 14)	*R* (*N* = 16)	p.overall
Cancers			0.058
ESCC	2 (14.3%)	8 (50.0%)	—
GC	12 (85.7%)	8 (50.0%)	—
Gender			1.000
Female	4 (28.6%)	4 (25.0%)	—
Male	10 (71.4%)	12 (75.0%)	—
Liver metastasis:			1.000
No	8 (57.1%)	10 (62.5%)	—
Yes	6 (42.9%)	6 (37.5%)	—
Number of metastatic organs			0.411
≤ 1	4 (28.6%)	8 (50.0%)	—
≥ 2	10 (71.4%)	8 (50.0%)	—
Age	55.5 (11.4)	59.2 (9.99)	0.358
BMI	22.4 (2.76)	21.3 (3.80)	0.374
PFS_group:			0.046
< 6 m	7 (50.0%)	2 (12.5%)	—
> 6 m	7 (50.0%)	14 (87.5%)	—

Initially, we measured the blood microbial diversity in patients with GI cancers using different methods (Shannon, observed species, Chao1, and PD whole tree indices) in the discovery set (Table 1). Species accumulation curves indicated that sequencing depth adequately covered species information in all groups, and data volume met analysis requirements (Figure S1A). We found that the alpha diversity of the tumor microbiome was significantly lower in the R group than in the NR group of patients receiving immunotherapy combined with chemotherapy. However, no significant difference was observed in patients who received chemotherapy alone (*p* < 0.05 for immunotherapy combined with chemotherapy and *p* > 0.05 for chemotherapy, Figure 1B). Next, we stratified the patients based on the median values of Shannon, observed species, and Chao1 indices and investigated the relationship between baseline tumor microbial diversity and progression‐free survival (PFS) in patients with GI cancers. As expected, we found that patients with lower baseline observed species and Chao1 indices had significantly prolonged PFS (*p* = 0.0015 for observed species and *p* = 0.013 for Chao1, Figure 1C), and patients with a lower Shannon index also showed a trend towards increased PFS (*p* = 0.074, Figure 1C) in those who received immunotherapy combined with chemotherapy. In contrast, no significant correlation was observed between baseline alpha diversity and PFS in patients receiving chemotherapy alone (*p* = 0.19 for Shannon, *p* = 0.53 for observed species, and *p* = 0.48 for Chao1, Figure 1C). Additionally, we analyzed the posttreatment alpha diversity index and found that the Chao1 index was significantly higher in the R group compared to the NR group after chemotherapy. However, no significant findings were observed in patients receiving immunotherapy combined with chemotherapy (Figure S1B).

### 2.2 Significant Differences in Blood Microbiota at the Genus and Phylum Levels Before and After Treatment in Immunotherapy Combined With Chemotherapy Patients

To explore the mutual impact between blood microbiota and different treatments (chemotherapy alone and immunotherapy combined with chemotherapy) in patients with GI cancers, we further analyzed the differences in blood microbiota communities before and after treatment and the association between beta diversity at the phylum and genus levels and treatment efficacy.

**FIGURE 2:**
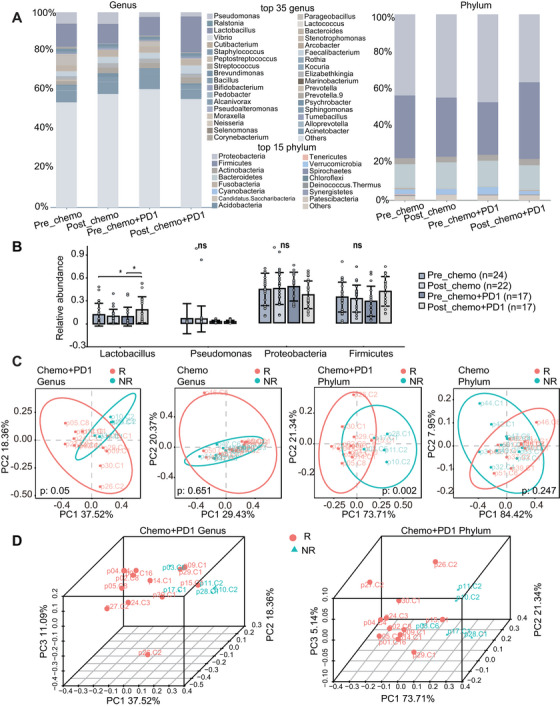
Significant differences in blood microbial community at genus and phylum levels before and after immunotherapy combined with chemotherapy. (A) Bar plots showing the relative abundance of microbial communities at the genus and phylum levels across four groups. (B) Bar plot illustrating dynamic changes in the relative abundance of *Lactobacillus*, *Pseudomonas*, *Proteobacteria*, and *Firmicutes* before and after treatment. (C) Principal Coordinates Analysis (PCoA) of beta diversity at genus and phylum levels, using Bray–Curtis distance metrics, comparing samples after chemotherapy and immunotherapy combined with chemotherapy. (D) 3D PCoA plots showing beta diversity at the genus (left) and phylum (right) levels using Bray–Curtis distance metrics, comparing samples after immunotherapy combined with chemotherapy. See also Figure S1.

We focused on analyzing the predominant phylum and genus in the blood samples of different groups. The top five phyla were *Proteobacteria, Firmicutes, Actinobacteria, Bacteroidetes*, and *Fusobacteria* (Figure 2A). *Proteobacteria* were the dominant phylum across all groups, with minimal changes before and after chemotherapy but a decreasing trend after immunotherapy combined with chemotherapy. However, *Firmicutes* showed an opposite increasing trend to *Proteobacteria* (Figure 2B). At the genus level, the top five genera with higher abundance in different group blood samples were *Pseudomonas*, *Ralstonia*, *Lactobacillus*, *Vibrio*, and *Cutibacterium* (Figure 2A). *Lactobacillus* was the dominant genus in the chemotherapy and immunotherapy combined with chemotherapy group, with minimal changes before and after chemotherapy, but a significant increase after immunotherapy combined with chemotherapy. *Pseudomonas* remained relatively stable before and after both therapy modalities, but it was still the main bacterial genus in these groups (Figure 2B).

To further analyze the relationship between blood microbiome diversity at the phylum and genus levels and the efficacy of immunotherapy, we performed beta diversity analysis at the phylum and genus levels. Principal Coordinates Analysis (PCoA) using Bray–Curtis distances showed no significant clustering differences between the R and NR groups before therapy (Figure S1C). However, the analysis after treatment indicated a significant clustering difference between the R and NR groups among patients receiving immunotherapy combined with chemotherapy (*p* = 0.05 at the genus level and *p* = 0.002 at the phylum level, Figure 2C), while no such differences were observed after chemotherapy alone (*p* = 0.651 at th0065 genus level and *p* = 0.247 at the phylum level, Figure 2C). Consistent results after treatment were obtained from 3D PCoA analyses of patients receiving both treatment modalities (Figure 2D and Figure S1D).

### 2.3 Patients With Low Relative Abundance of Blood *Lactobacillus* at Baseline Benefit More From Immunotherapy Combined With Chemotherapy Compared to Chemotherapy Alone

Given the significant differences in the proportion of *Lactobacillus* before and after immunotherapy combined with chemotherapy, as well as the observed trends in *Proteobacteria* and *Firmicutes*, we further investigated their impact on PFS in patients receiving immunotherapy combined with chemotherapy or chemotherapy alone. Additionally, due to the high relative abundance of *Pseudomonas* at the genus level, we conducted an in‐depth analysis of *Pseudomonas*.

**FIGURE 3:**
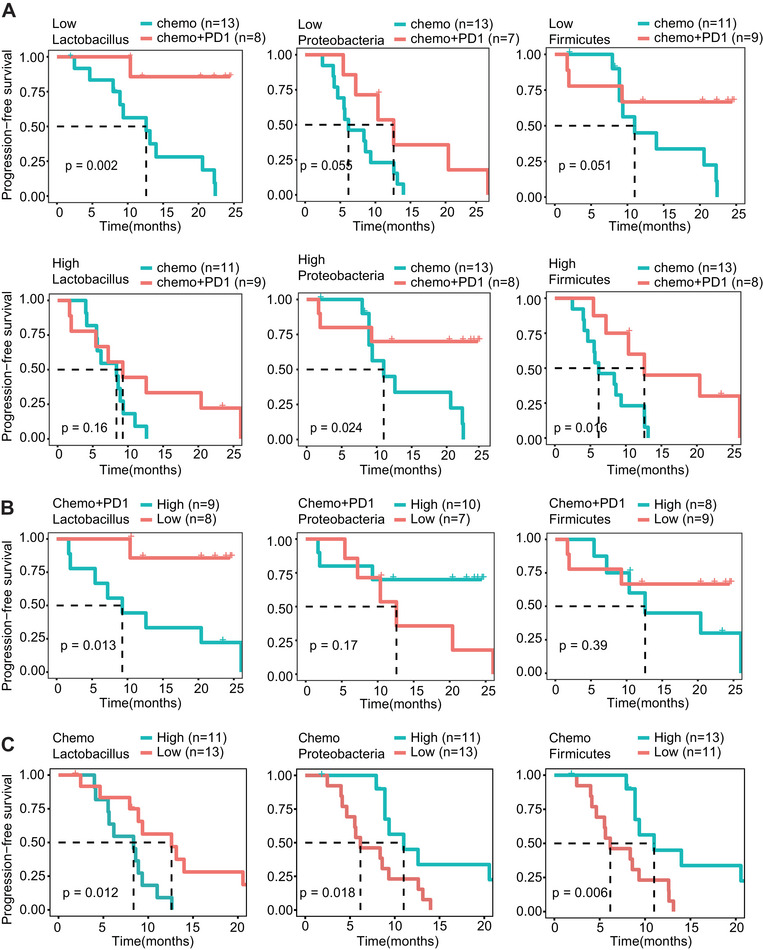
Low baseline abundance of *Lactobacillus* is associated with longer progression‐free survival (PFS). (A) Kaplan–Meier survival curves of gastrointestinal cancer patients before chemotherapy (*n* = 24) and immunotherapy combined with chemotherapy (*n* = 17), categorized by the relative abundance of *Lactobacillus*, *Proteobacteria*, and *Firmicutes*. (B) Kaplan–Meier survival curves of gastrointestinal cancer patients before immunotherapy combined with chemotherapy (*n* = 17), categorized by the relative abundance of *Lactobacillus*, *Proteobacteria*, and *Firmicutes*. (C) Kaplan–Meier survival curves of gastrointestinal cancer patients before chemotherapy (*n* = 24), categorized by the relative abundance of *Lactobacillus*, *Proteobacteria*, and *Firmicutes*. See also Figure S2.

Patients were stratified based on baseline blood microbiota (*Lactobacillus, Pseudomonas, Proteobacteria*, and *Firmicutes*) relative abundance, using the median relative abundance as cutoff values (*Lactobacillus*: 0.0425, *Pseudomonas*: 0.02, *Proteobacteria*: 0.4488, and *Firmicutes*: 0.3088). Patients were classified into the high‐abundance blood microbiota group and the low‐abundance blood microbiota group based on the cutoff. It was found that at the genus level, patients with lower relative abundance of *Lactobacillus* (≤ 0.0425) and *Pseudomonas* (≤ 0.02) benefited more from immunotherapy combined with chemotherapy compared to chemotherapy alone (*p* = 0.0011 for *Lactobacillus* and *p* = 0.0019 for *Pseudomonas*, Figure 3A and Figure S2A). Conversely, patients with higher relative abundance of *Lactobacillus* (> 0.0425) and *Pseudomonas* (> 0.02) did not exhibit significant differences in PFS between immunotherapy combined with chemotherapy and the chemotherapy group (*p* = 0.67 for *Lactobacillus* and *p* = 0.28 for *Pseudomonas*, Figure 3A and Figure S2A). Meanwhile, at the phylum level, we found that *Proteobacteria* and *Firmicutes* exhibited longer PFS in patients receiving immunotherapy combined with chemotherapy compared to chemotherapy alone (Figure 3A). These results reflect that the relative abundance of blood *Lactobacillus* and *Pseudomonas* may serve as biomarkers to predict the benefit from immunotherapy combined with chemotherapy compared to chemotherapy alone.

To identify the blood microbiota most strongly associated with PFS in patients receiving immunotherapy combined with chemotherapy, we separately analyzed the impact of these four blood microbiota on PFS in both therapy modalities. We found that only blood *Lactobacillus* was significantly associated with PFS in both therapies (Figure 3B,C), while the other three microbiota were significantly associated with PFS only in patients receiving chemotherapy (Figure 3B,C and Figure S2A). We also analyzed the relationship between these major microbiota and treatment efficacy. Consistent to some extent with PFS, the baseline blood *Lactobacillus* abundance was higher in NR patients compared to R patients, although no statistical difference was found (Figure S2B).

These results show that patients with a lower baseline relative abundance of blood *Lactobacillus* exhibited longer PFS after immunotherapy combined with chemotherapy, suggesting that baseline blood *Lactobacillus* relative abundance could serve as a potential biomarker for predicting the efficacy of immunotherapy combined with chemotherapy in GI cancer patients.

### 2.4 Validation of Baseline Blood *Lactobacillus* as a Biomarker for Predicting the Efficacy of Immunotherapy Combined With Chemotherapy

To further investigate the correlation between baseline blood *Lactobacillus* abundance and PFS in GI cancer patients receiving immunotherapy combined with chemotherapy, we added an additional cohort of patients for validation. PBMCs from GI cancer patients (Table 2, Validation Set 1) were collected before and after immunotherapy combined with chemotherapy for 16S rRNA sequencing, totaling 84 samples, including 33 paired samples (Figure 1A). Species accumulation curves indicated that sequencing depth adequately covered species information in all groups, and data volume met analysis requirements (Figure S2C).

**FIGURE 4:**
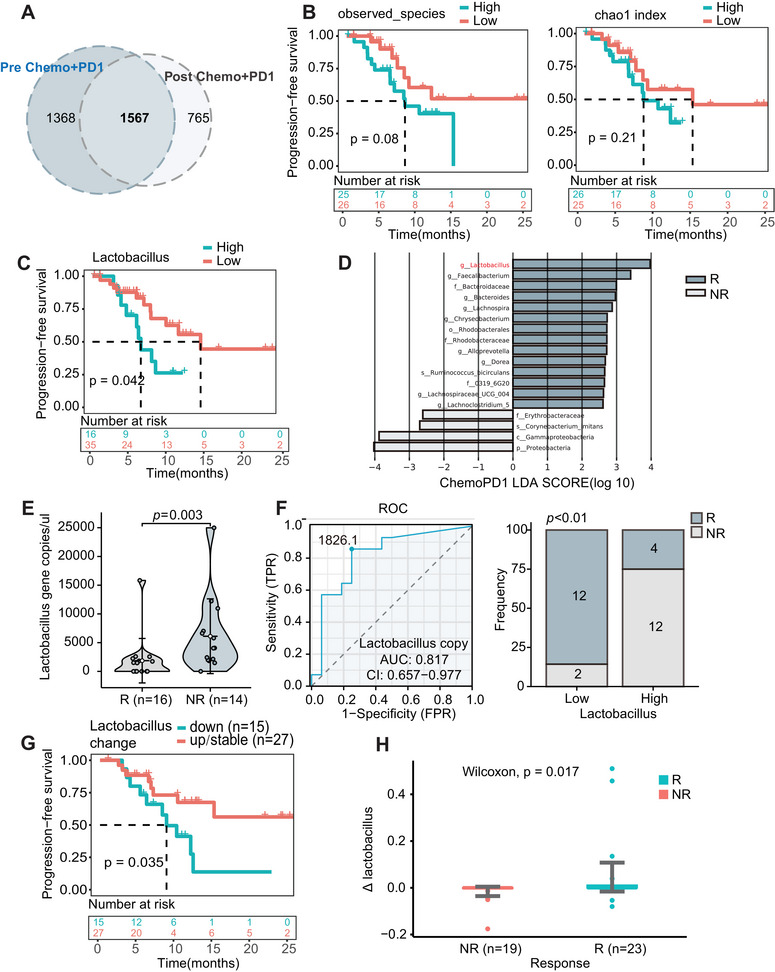
Baseline abundance and dynamic changes of *Lactobacillus* influence the response to immunotherapy combined with chemotherapy. (A) Venn diagram depicting the difference of blood microbiome communities before and after immunotherapy combined with chemotherapy (Validation Set 1, before and after treatment, *n* = 84). (B) Kaplan–Meier survival curve of gastrointestinal cancer patients categorized by alpha diversity indices (observed species and Chao1 index) before immunotherapy combined with chemotherapy (Validation Set 1, pretreatment, *n* = 51). (C) Kaplan–Meier survival curve stratifying patients with gastrointestinal cancer based on the relative abundance of *Lactobacillus* before immunotherapy combined with chemotherapy (Validation Set 1, pretreatment, *n* = 51). (D) Linear discriminant analysis (LDA) score showing features with differential abundance in R and NR groups after chemotherapy combined with immunotherapy (LDA score > 2.6). (E) Violin plots of *Lactobacillus* gene copy numbers in R and NR groups during immunotherapy combined with chemotherapy (Validation Set 2, *n* = 30). (F) ROC analysis of *Lactobacillus* gene copy numbers as a predictor of therapeutic efficacy (Validation Set 2, *n* = 30). (G) Kaplan–Meier survival analysis of gastrointestinal cancer patients, defined by dynamic changes in *Lactobacillus*. A total of 42 paired samples before and after treatment are included in both the discovery set and Validation Set 1. (H) Box plot illustrating dynamic changes in the relative abundance of *Lactobacillus* before and after treatment. The *y*‐axis represents the value of posttreatment and pretreatment differences.

Firstly, we analyze the changes in blood microbiota composition before and after treatment, observing substantial alterations in the blood microbiome following immunotherapy combined with chemotherapy, highlighting the impact of this treatment on the blood microbiota composition in GI cancer patients (Figure 4A). We then validated the correlation between PFS and blood microbiome diversity in Validation Set 1. As expected, patients with a lower alpha diversity index showed a trend toward increased PFS after receiving immunotherapy combined with chemotherapy (*p* = 0.08 for observed species and *p* = 0.21 for Chao1, Figure 4B). Furthermore, stratification based on baseline blood *Lactobacillus* relative abundance in Validation Set 1 revealed that, consistent with expectations, patients with lower baseline *Lactobacillus* abundance experienced significantly improved PFS following combined immunotherapy and chemotherapy (cutoff defined by the surv_cutpoint function in R, *p* = 0.042, Figure 4C). Linear discriminant analysis effect size (LEfSe) was used to identify significantly different microbial taxa between the R and NR groups after treatment, with a threshold LDA score > 2.6. We found that *Lactobacillus* was the most prominent marker in the R group among patients receiving combined immunotherapy and chemotherapy (Figure 4D).

Subsequently, we collected pretreatment PBMC samples from 30 patients undergoing immunotherapy combined with chemotherapy (Validation Set 2). The *Lactobacillus* gene copy number in the blood was quantified using absolute bacterial quantification via quantitative polymerase chain reaction (qPCR). Clinical information is provided in Table 3. As expected, patients with response had significantly lower baseline *Lactobacillus* copy numbers compared with patients with nonresponse from immunotherapy combined with chemotherapy (*p* = 0.003, Figure 4E). Receiver operating characteristic (ROC) curve analysis indicated that the baseline *Lactobacillus* copy number could predict the efficacy of immunotherapy combined with chemotherapy, with an area under the curve (AUC) of 0.817 and an optimal cutoff value of 1826.1 (Figure 4F left). Based on the optimal cutoff, patients were stratified into high *Lactobacillus* copy number (*Lactobacillus* > 1826.1 copies/uL) and low *Lactobacillus* copy number (*Lactobacillus* ≤ 1826.1 copies/uL) groups. We found that patients with lower baseline *Lactobacillus* copy numbers had a significantly higher proportion of response to immunotherapy combined with chemotherapy compared to patients with higher baseline *Lactobacillus* copy numbers (85.7% vs. 25.0%, *p* < 0.01, Figure 4F right).

### 2.5 Increased *Lactobacillus* Abundance in Blood After Immunotherapy Combined With Chemotherapy Predicts Longer PFS

To explore the relationship between dynamic changes in *Lactobacillus* and immunotherapy combined with chemotherapy, we integrated data from 16S rRNA data from the discovery set (*n* = 17) and Validation Set 1 (*n* = 51) to analyze the relationship between changes in *Lactobacillus* abundance and PFS in patients undergoing immunotherapy combined with chemotherapy. We found that patients with stable or increased posttreatment *Lactobacillus* relative abundance had significantly superior PFS (*p* < 0.05, Figure 4G). Further analysis of treatment efficacy indicated that the change from baseline after immunotherapy combined with chemotherapy in the R group was significantly greater than in the NR group, consistent with the results of the PFS analysis (Figure 4H).

The result confirmed the correlation between baseline *Lactobacillus* relative abundance, dynamic changes, and immunotherapy combined with chemotherapy, suggesting that blood *Lactobacillus* abundance and its dynamics may serve as predictive indicators for the efficacy of immunotherapy combined with chemotherapy in GI cancers.

### 2.6 Synergistic Antitumor Effect of Elevating *Lactobacillus rhamnosus* Blood Level by Gavage Combined With PD‐1 in an In Vivo Model

**FIGURE 5:**
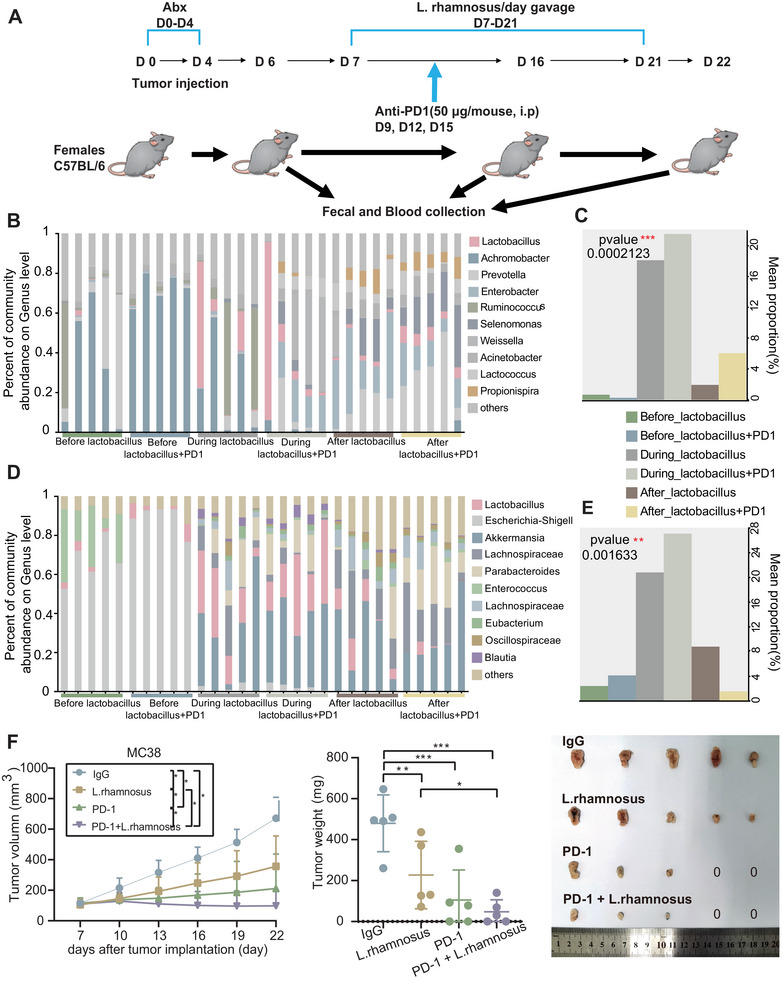
Development of a subcutaneous transplant tumor model in a mouse. (A) Flowchart of the experimental procedures in mice (*n* = 20). (B) Bar chart illustrating the genus‐level composition of blood microbiota in mice at different time points during bacterial inoculation. (C) Bar chart showing the relative abundance of *Lactobacillus* in the blood of mice at different time points during bacterial inoculation. (D) Bar chart illustrating the genus‐level composition of fecal microbiota in mice at different time points during bacterial inoculation. (E) Bar chart showing the relative abundance of *Lactobacillus* in the feces of mice at different time points during bacterial inoculation. (F) Plot showing differential tumor growth curves during *Lactobacillus rhamnosus* gavage, along with a comparison of tumor weights at the endpoint. See also Figure S3.

To apply our findings about *Lactobacillus rhamnosus* (*L. rhamnosus*) in the synergistic enhancement of anti‐PD‐1 treatment effect in vivo, we established a subcutaneous colon cancer mouse model. We evaluated the antitumor effect of elevating *L. rhamnosus* blood levels by gavage in combination with PD‐1 antibody by observing tumor changes in mice following this combined intervention (Figure 5A).

We measured the relative abundance of *Lactobacillus* in the blood and feces of mice at different time points. Species accumulation curves indicated that sequencing depth adequately covered species information in all groups, and data volume met analysis requirements (left for blood and right for fecal, Figure S3A). We found that the blood and fecal *Lactobacillus* levels were initially very low but increased significantly after gavage (Figure 5B,D). Analysis of similarity (ANOSIM) was performed to evaluate the statistical difference between the microbiome compositions. We found a significant difference in overall tumor microbiome composition between before *Lactobacillus* and after *Lactobacillus* groups (*p* = 0.001 and *R* = 0.6322 for blood, *p* = 0.001 and *R* = 0.7537 for fecal, Figure S3B,C). As expected, the PD‐1 plus *L. rhamnosus* group showed higher blood *Lactobacillus* levels after treatment compared to the *L. rhamnosus* group (Figure 5C). Conversely, the fecal *Lactobacillus* abundance in the PD‐1 plus *L. rhamnosus* group was significantly lower than in the *L. rhamnosus* group (Figure 5E), likely due to increased *Lactobacillus* absorption into the bloodstream after PD‐1 antibody treatment.

Mice treated with *L. rhamnosus* during anti‐PD‐1 treatment had elevated blood levels of *L. rhamnosus* and exhibited significantly slower tumor growth compared to the IgG control group (*p* < 0.05, Figure 5F). Additionally, mice receiving the combination of *L. rhamnosus* and PD‐1 antibody showed even greater inhibition of tumor growth and significantly smaller tumor volumes compared to all other groups (*p *< 0.05, Figure 5F). The antitumor efficacy of the *L. rhamnosus* and PD‐1 combination was superior to that of PD‐1 monotherapy, indicating a synergistic antitumor effect of the combined treatment.

At the end of the study, tumors in the *L. rhamnosus* group were smaller than in the IgG control group, while tumors in the PD‐1 monotherapy and PD‐1 plus *L. rhamnosus* groups were smaller compared to the IgG control and *L. rhamnosus* groups (Figure 5F). Some mice in these groups showed complete tumor regression. Furthermore, mice in the PD‐1 plus *L. rhamnosus* group had smaller tumors compared to those receiving PD‐1 alone. Tumor weight in the L. rhamnosus, PD‐1 monotherapy, and PD‐1 plus *L. rhamnosus* groups was significantly lighter compared to the IgG control group (*p* < 0.01, Figure 5F). Additionally, tumor weight was lower in the PD‐1 plus *L. rhamnosus* group compared to the *L. rhamnosus* group (*p* < 0.05, Figure 5F), and although the median tumor weight was lower in the PD‐1 plus *L. rhamnosus* group compared to the PD‐1 monotherapy group, the difference was not statistically significant (*p* > 0.05, Figure 5F).

In summary, we used a subcutaneous colon cancer mouse model to demonstrate that the combination of *L. rhamnosus* gavage and PD‐1 antibody yields a synergistic antitumor effect, as evidenced by differences in tumor growth, anatomical features, and weight, along with changes in *Lactobacillus* abundance in the blood and feces.

## 3 Discussion

Immunotherapy has revolutionized the treatment of GI cancers, offering new hope where traditional therapies fall short [29]. Despite significant advances, current biomarkers for predicting immunotherapy efficacy in GI cancers remain unreliable, highlighting the need for new predictive tools [16].

The human microbiome, particularly the gut microbiome, has emerged as a critical player in modulating immune responses and the efficacy of immunotherapy. Recent studies suggest that the composition of the microbiome can modulate TME, oncogenesis, metastatic potential, and the host's immune response [22, 30, 31], making it a potential biomarker for predicting immunotherapy outcomes. Emerging evidence suggests potential mechanistic links between the microbiome and immune response in cancers. While direct studies on microbiome and established biomarker (e.g., PD‐L1 expression, MSI status) interplay remain limited, insights can be extrapolated from related niches: Gut microbiome‐derived butyrate suppresses PD‐L1 and IL‐10 expression in tumor‐associated macrophages, thereby enhancing antitumor immunity in gastric cancer [32], and in lung cancer, higher diversity of the lung microbiome correlates with elevated PD‐L1 expression and improved response to anti‐PD‐1 therapy [33]. However, the absence of direct evidence in the blood microbiome in GI cancers underscores a critical knowledge gap. This offers a promising approach for developing more effective and personalized microbiome‐targeted therapies, which can modify the tumor microenvironment to enhance immunotherapy or prevent resistance.

Our study found that the blood microbiota community is significantly different before and after therapy in patients with immunotherapy combined with chemotherapies, while in patients who received chemotherapies alone, the blood microbiota community was relatively stable. This indicated that immunotherapy, which is designed to unleash the immune system to attack cancer cells, can affect the immune system and lead to changes in microbial diversity and composition. And we compared the blood microbial diversity between the response patients and the nonresponse patients in the chemoPD1 group; we found that the baseline alpha diversity was significantly lower in responders than in nonresponders. Notably, increased alpha diversity was correlated with improved outcome in early studies [34]. Nevertheless, evidence for the exact association between microbial diversity and the ICI effectiveness is lacking. The varied study results can be explained by the different sources of microbiomes, either from the blood, the gut, or the tumor itself. Moreover, the specific microbiomes associated with different tumors themselves may respond differently to immunotherapy [35].

Initial analysis at the genus and phylum levels suggests that blood levels of *Lactobacillus*, *Pseudomonas*, *Proteobacteria*, and *Firmicutes* may serve as candidate biomarkers for predicting the efficacy of immunotherapy. Further stratified analysis based on baseline microbiome abundance in the discovery set reveals that only the abundance of *Lactobacillus* is significantly associated with PFS in patients undergoing immunotherapy combined with chemotherapy. Subsequent findings from Validation Set 1 and Validation Set 2 support this observation. These results indicate that blood *Lactobacillus* not only serves as a suitable candidate biomarker for selecting patients with GI cancer for immunotherapy combined with chemotherapy but also as a novel biomarker for predicting treatment outcomes. Furthermore, our analysis suggests that dynamically monitoring the blood abundance of *Lactobacillus* can provide valuable guidance for adjusting treatment plans during the course of therapy. This conclusion underscores the important role of *Lactobacillus* in the antitumor immune response of GI cancer patients undergoing immunotherapy. These findings are also consistent with previous research on the role of *Lactobacillus* in tumor immunity.

While the gut microbiome has emerged as a well‐characterized predictor of immunotherapy response across malignancies, its comparative performance against blood‐derived microbial biomarkers remains underexplored. Insights can be drawn from cross‐cancer studies: A study in NSCLC demonstrated that PFS following anti‐PD‐1 therapy could be optimally predicted by gut microbiome signatures, achieving an AUC of 0.74 [36], while a pan‐cancer analysis identified four conserved gut microbial functional pathways from fecal metagenomes that predicted immunotherapy response with an AUC of 0.81 via random forest modeling [37]. Although these findings cannot be directly extrapolated and compared to blood microbiome predictive capacities, our study demonstrated comparable predictive performance (AUC = 0.817), suggesting blood microbiome biomarkers may also exhibit competitive advantages in specific clinical contexts. Future studies should prioritize multi‐omics integration (e.g., paired blood microbiome and fecal microbiome) in large prospective cohorts to delineate niche‐specific predictive capacities.

Numerous studies have explored the connection and interactions between *Lactobacillus* microbiomes, immunity, and cancer. *Lactobacillus* are positively correlated with antitumor immune factors [38, 39]. *Lactobacillus* is a probiotic strain that can stimulate host immune function and prevent intestinal inflammation and infection [40]. *Lactobacillus* HDB1258 isolated from the feces of breastfed infants can play an antitumor effect by activating innate immunity to enhance the immune response, including significantly increasing the cytotoxicity of NK cells and the phagocytosis of macrophages [41]. *Lactobacillus johnsonii* can significantly improve the efficacy of ICI in four cancer mouse models [42]. In a mouse CRC model, Sini Decoction can increase the expression of CD8^+^ T lymphocytes and decrease CD4+ T cells and inflammatory cytokine levels, effectively intervening in cancer progression. This effect may be associated with an increased abundance of intestinal Lactobacillus [43]. These study findings suggest that increasing the levels of *Lactobacillus* may enhance the effectiveness of immunotherapy. Thus, we constructed a subcutaneous transplant tumor model in mice, and we observed that tumors grew the slowest and had the smallest weight when PD‐1 was combined with *Lactobacillus*. This suggests that *Lactobacillus* has the potential to act as a sensitizer for immunotherapy, although further research is needed to confirm this.

Currently, immunotherapy for GI cancers lacks novel and effective biomarkers to predict efficacy. Although the blood microbiome is a cutting‐edge topic, there are few studies on the blood microbiome in the context of immunotherapy for GI cancers. Our research partially fills this gap, providing important evidence for the precision selection of PD‐1‐responsive patients and the development of combined strategies to enhance PD‐1 therapy efficacy. This study offers options for the development of blood microbiome biomarkers and therapeutic targets for GI cancer immunotherapy, with broad application prospects.

However, our study still has limitations and several issues that need to be addressed. As shown by the study results above and previous studies, *Lactobacillus* is mostly considered a probiotic in the body, exerting antitumor effects by regulating the immune response through various metabolic products or other mechanisms. This raises the question: why do patients with lower baseline Lactobacillus levels show better outcomes than those with higher levels? One possible reason is that *Lactobacillus* comprises different strains, each of which has different effects on the immune system [44]. Some strains can induce an increase in IL‐10 levels, a key immunosuppressive cytokine in the human body [41, 45, 46, 47]. Our study only measured the overall levels of *Lactobacillus* without analyzing specific strains. If the strains that elevate IL‐10 levels are present at relatively low levels, this could potentially explain why these patients respond better to immunotherapy. Alternatively, it could also be due to the effects of other specific *Lactobacillus* strains. In summary, the underlying mechanisms remain to be elucidated. The second limitation is that, although we demonstrated that the combination of *Lactobacillus* and PD‐1 leads to a greater reduction in tumor volume, we did not explore the underlying mechanisms. Previous studies have shown that *L. rhamnosus* GG can limit tumor progression by reducing colorectal cell invasion through influencing MMP‐9 activity [48]. Our experimental results also suggest that this strain can work synergistically with immunotherapy to inhibit tumor growth. However, whether additional immunomodulatory mechanisms are involved still requires further experimental investigation. Additionally, the sample size in our validation cohort was relatively small, resulting in some findings lacking statistical significance; more samples may be needed in future studies for validation.

## 4 Conclusion

In conclusion, our study indicates that blood microbiota can be detected in GI cancer patients, and it shows significant potential as a novel biomarker for predicting the efficacy of immunotherapy. Our analysis also revealed that patients with a lower baseline relative abundance of *Lactobacillus* benefited more from immunotherapy combined with chemotherapy than from chemotherapy alone, suggesting that this characteristic could be used in the future to select patients for immunotherapy. Moreover, patients with lower alpha diversity, lower copy numbers of *Lactobacillus*, and those whose relative abundance of *Lactobacillus* remained stable or increased after treatment also benefited more from immunotherapy combined with chemotherapy. This suggests that these indicators could potentially guide clinical treatment choices. Our study, using a mouse subcutaneous tumor model, found that *Lactobacillus* combined with PD‐1 immunotherapy has a synergistic antitumor effect, laying an experimental foundation for its future therapeutic applications.

## 5 Materials and Methods

### 5.1 Patients and Samples

The study was conducted at the Sun Yat‐Sen University Cancer Center (SYSUCC) from January 2019 until May 2022 and recruited 134 patients with stage III or stage IV GI cancers confirmed by histology or cytology, including 52 esophageal cancer patients, 65 gastric cancer patients, and 17 CRC patients. This retrospective study was approved by the Sun Yat‐Sen University Cancer Center Ethics Committee (B2022‐166‐01, Guangdong, China). All enrolled patients signed an informed consent form for specimen collection prior to blood sampling. PBMCs were collected from these patients, and microbial DNA was analyzed using 16S rRNA and qPCR. The composition of microbial DNA and its correlation with immunotherapy efficacy were assessed. A total of 164 samples were obtained from these patients before and after treatment, including 60 paired 16S rRNA samples collected before and after treatment. For first‐line therapy, there were 106 patients receiving chemotherapy combined with anti‐PD‐1 immunotherapy, while 28 patients received chemotherapy alone. Patients received the treatment until disease progression or intolerable toxicity. The Response Evaluation Criteria in Solid Tumors (RECIST) version 1.1 were used to assess treatment response utilizing CT‐based monitoring every 6–8 weeks. R were those who had a complete response (CR) or a partial response (PR) as their best response, whereas NR had stable disease (SD) or progressing disease (PD). PFS was calculated from the date of commencement of treatment until the date of verification of progressive disease by CT scan.

### 5.2 Cell Lines and Cell Culture Conditions

MC‐38 cell lines were obtained from the American Type Culture Collection (ATCC, USA). MC‐38 cells were grown in DMEM medium containing 10% fetal bovine serum. All cells were maintained at 37°C in a humidified 5% CO_2_ incubator.

### 5.3 Animals

C57BL/6 mice (*n* = 20) were purchased from Guangdong Vital River Laboratory Animal Technology Co. Ltd., and kept in a specific pathogen‐free (SPF) barrier facility at the Animal Center of Sun Yat‐Sen University Cancer Center. 8–12‐week‐old female mice were used for all animal experiments. Experiments were approved by the institutional committee of Sun Yat‐Sen University Cancer Center (No. L025504202206014) and conducted following protocols approved by the Guangdong Provincial Animal Care and Use Committee. The *L. rhamnosus* used in this study was isolated from the feces of patients with GI cancers who showed favorable immune responses to immunotherapy, and the live bacteria were provided by the Guangdong Institute of Microbiology (preservation center number: GDMCC 1.3426).

Mice were treated with an antibiotic solution (ATB) containing ampicillin (1 mg/mL), streptomycin (5 mg/mL), and colistin (1 mg/mL) (Thermo Fisher Scientific), with the addition of vancomycin (0.25 mg/mL) via gavage for 5 days. MC38 cells (2 × 106) were subcutaneously injected in the right flank of mice. Mice were pooled and randomly divided into different groups when the tumor reached a volume of approximately 100 mm^3^. IgG isotype control antibody and anti‐PD‐1 antibody (50 µg per mouse) were injected intraperitoneally on Days 9, 12, and 15. *L*. *rhamnosus* was orally administered on Days 7–21. Tumors were measured every 3 days. Animals were euthanized when the tumor volume reached 2000 mm^3^. Survival analysis was performed using Kaplan–Meier analysis and the log‐rank test.

### 5.4 Blood Microbiome and Feces Microbiome

PBMCs were isolated from diluted human blood using density gradient centrifugation over Ficoll‐Paque (MD PACIFIC Biotech, Tianjin, China). The isolated cells were washed twice with saline, collected, and stored at −80°C until DNA extraction. Blood and fecal samples from mice were stored at −80°C until DNA extraction.

The variable regions (V3–V4) of the 16S rRNA gene were sequenced to assess the blood and fecal microbiomes. PCR amplification of the V3–V4 region of the bacterial 16S rRNA gene was performed using primers 341F and 806R. Sequencing libraries were prepared using the Illumina TruSeq DNA PCR‐Free Library Preparation Kit (Illumina, USA) following the manufacturer's recommendations, and index codes were added for sample identification. The library quality was assessed on the Qubit 2.0 Fluorometer (Thermo Fisher Scientific) and Agilent Bioanalyzer 2100 system. At last, the library was sequenced on an Illumina NovaSeq6000 platform, and 250 bp paired‐end reads were generated. Paired‐end reads were assigned to each sample according to the unique barcodes. The reads were demultiplexed, trimmed, and filtered to remove low‐quality reads using the QIIME (v1.17) software. The high‐quality, paired‐end reads with overlap were merged into tags by using FLASH. The criterion of overlapping was the overlapping region lengths larger than 10 bp and a mismatch ratio lower than 0.2. Chimera tags were removed using UCHIME (v4.2.40) against the Gold database (v13.8). The remaining sequences were clustered into operational taxonomic units (OTUs) at 97% sequence similarity using VSEARCH (v2.4.4). The representative sequences for each OUT were taxonomically classified using RDP Classifier (v.2.2) trained on the Silva database (v132) by Mothur software. OTUs were processed by removing chloroplast, mitochondrial, and unclassified sequences using VSEARCH. The OTU table was rarefied by using the “multiple_rarefactions.py” script in QIIME. This OTU table was then used to calculate the alpha diversity and beta diversity and to provide taxonomic profiles.

### 5.5 Quantitative Polymerase Chain Reaction

Genomic DNA was extracted using the TSINGKE plant DNA extraction kit (TSINGKE, China) following the manufacturer's protocol. Samples of dry tissue (≤ 20 mg) were ground in liquid nitrogen and treated with a series of buffers to lyse the cells and purify the DNA. The purified DNA was eluted in TE buffer and stored at −20°C for further analysis. Each reaction contained 10 µL of qPCR mix, 0.7 µL of forward and reverse primers (10 µM each), 0.6 µL of probe (10 µM), 1 µL of DNA template, and water to a final volume of 20 µL. The thermal cycling conditions were 95°C for 1 min, followed by 40 cycles of 95°C for 15 s and 60°C for 30 s. Fluorescence data were collected at 60°C. The mRNA expression levels of target genes were quantified using the 2^−△△^ *^C^* ^t^ method. Primer sequences for the real‐time qPCR are shown in Table S1.

### 5.6 Statistical Analysis

Categorical data between two or multiple groups were compared using a Wilcoxon test. PFS and response to therapy were designated as the primary efficacy indicators for the biomarker analysis. Statistical analyses were performed using R (version 4.2.2). Survival analysis was performed using Kaplan–Meier curves and evaluated with the log‐rank test. Alpha diversity indices, including the Shannon index for diversity, the Chao1 index for richness, and Observed Species and Phylogenetic Distance (PD_whole), were calculated from rarefied tables using the “alpha_diversity.py” script in QIIME. Rarefaction curves of the four alpha diversity indices were calculated at a depth of 30,000 reads. The Bray–Curtis distance, calculated based on the relative abundance at the genus and phylum levels, was used to assess the microbial beta‐diversity between groups. PCoA plots were used to evaluate differences in microbial community composition and identify potential clusters. Differences in microbial community structure were evaluated using nonparametric multivariate analysis methods, ANOSIM. Utilizing R software, Venn diagrams were generated indicating specific and common OTUs amongst pretreatment and posttreatment. LEfSe was used to identify the microbial taxa that significantly differed between the R and NR groups, with a threshold LDA score of > 2.6 used.

The data generated from the mouse model were analyzed by GraphPad Prism (V.8.0) software. Unpaired Student's *t*‐tests were used to determine statistical significance between two groups, with two‐tailed *p* values calculated. A two‐way analysis of variance (ANOVA) was performed when more than two groups were compared. All reported *p* values are two‐tailed. *P* values are labeled in the figures. *P* values were denoted as follows: *p < 0.05, **p < 0.01, ***p < 0.001.

## Author Contributions

Y.L., R.H.X., and Z.L.Z. conceived and designed the overall study. All authors were involved with the writing of the manuscript. Y.S.Z. and W.H.L. performed the data cleaning and bioinformatics analyses. Y.S.Z., W.H.L., J.Q.C., and X.L.W. performed sequencing and functional assays. Y.S.Z., W.H.L., J.Q.C., X.L.W., and J.Q.H. designed the experiments. J.Q.C., J.Q.H., and Y.H.X. constructed mouse models and contributed to the experimental design. X.L.W., J.Q.H., Y.H.X., M.Y., Q.H.Z., Z.J.Z., and Z.Y.Y. contributed to sample acquisition and clinical annotations. P.Z., N.K.H., and L.X.L. participated in the experiment and revised the article. H.Y.L., R.H.X., and Z.L.Z. supervised the study. All authors reviewed and approved the final manuscript.

## Conflicts of Interest

The authors declare no conflicts of interest.

## Ethics Statement

This retrospective study was approved by the Sun Yat‐Sen University Cancer Center (SYSUCC) Ethics Committee (B2022‐166‐01, Guangdong, China). All animal studies were approved by the institutional committee of Sun Yat‐sen University Cancer Center (No. L025504202206014).

## Supporting information




**Supplementary Table 1**: qPCR Primers sequences. **Supplementary Figure 1**: Blood microbial diversity and community structure before and after treatment. **Supplementary Figure 2**: Blood microbial abundance and survival analysis in gastrointestinal cancer patients. **Supplementary Figure 3**:Blood microbial diversity and community structure in mouse blood and fecal samples.

## Data Availability

Data are available in a public, open‐access repository. Data are available on reasonable request. The 16S rRNA sequencing data were deposited in the Mendeley repository (doi: 10.17632/zvcfjtschv.2).
