# Supplementary material for: Blood Microbiome Reveals the Impact of Lactobacillus on the Efficacy of Immunotherapy in Gastrointestinal Cancer
Source: MedComm (2020). 2025 Aug 11;6(8):e70316. doi: 10.1002/mco2.70316 (PMC12336454; doi:10.1002/mco2.70316)
Supplement: Supplementary file 1 — Supplementary Table 1: qPCR Primers sequences. Supplementary Figure 1: Blood microbial diversity and community structure before and after treatment. Supplementary Figure 2: Blood microbial abundance and survival analysis in gastrointestinal cancer patients. Supplementary Figure 3:Blood microbial diversity and community structure in mouse blood and fecal samples. [file MCO2-6-e70316-s001.pdf]

Title: Blood Microbiome Reveals the Impact of Lactobacillus on the Efficacy of Immunotherapy in Gastrointestinal Cancer

Authors and affiliations:

Ya-Shang Zheng[1,2,#], Wu-Hao Lin[3,#], Jun-Quan Chen[4,#], Xiao-Li Wei[1,2,#], Jia-Qian Huang[1,2], Yu-Hong Xu[1,2], Meng Yang[1,2], Qi-Hua Zhang[1,2], Zhi-Jun Zuo[1,2], Zhao-Ying Yang[1,2], Zhao-Lei Zeng[1,2*], Rui-Hua Xu[1,2,*], Hui-Yan Luo[1,2*]

[1]Department of Medical Oncology, State Key Laboratory of Oncology in South China, Collaborative Innovation Center for Cancer Medicine, Guangdong Provincial Clinical Research Center for Cancer, Sun Yat-sen University Cancer Center, Guangzhou, 510060, P. R. China.

[2]Research Unit of Precision Diagnosis and Treatment for Gastrointestinal Cancer, Chinese Academy of Medical Sciences, Guangzhou, 510060, P. R. China.

[3]Department of Molecular Diagnostics, State Key Laboratory of Oncology in South China, Collaborative Innovation Center for Cancer Medicine, Guangdong Provincial Clinical Research Center for Cancer, Sun Yat-sen University Cancer Center, Guangzhou 510060, China.

[4]Department of Anesthesiology, Sun Yat-sen University Cancer Center, State Key Laboratory of Oncology in South China, Collaborative Innovation Center for Cancer Medicine, 510060, Guangzhou, Guangdong, China.

[#]Ya-Shang Zheng, Wu-Hao Lin, Jun-Quan Chen, WuHao Lin, Xiao-Li Wei contributed equally.

[*]Correspondence to:

Hui-Yan Luo: luohy@sysucc.org.cn, Rui-Hua Xu: xurh@sysucc.org.cn, Zhao-Lei Zeng: zengzhl@sysucc.org.cn

Supplementary Figures and Tables

Table S1. qPCR Primers sequences	
**Primers**	**Sequence(5'to3')**
acinetobacter-F	CGTCAGTRTTAGGCCAGATGGCT
acinetobacter-R	TGTGAAATCCCYGAGCTTAACT
Burkholderia-F	ATGAAAGCGGGGGACCTTC
Burkholderia-R	CCCCACCAACTAGCTAATCAGC
Lactobacillus-F	GCGRTGCATTAGCTAGTTGGT
Lactobacillus-R	GGCCGTGTCTCAGTCCCA
acinetobacter-Probe	ATTGCATTCGATACTGG
Burkholderia-Probe	ATCGGCCAACCCTATAG
Lactobacillus-probe	TCAGTCTCTCAACTCGG

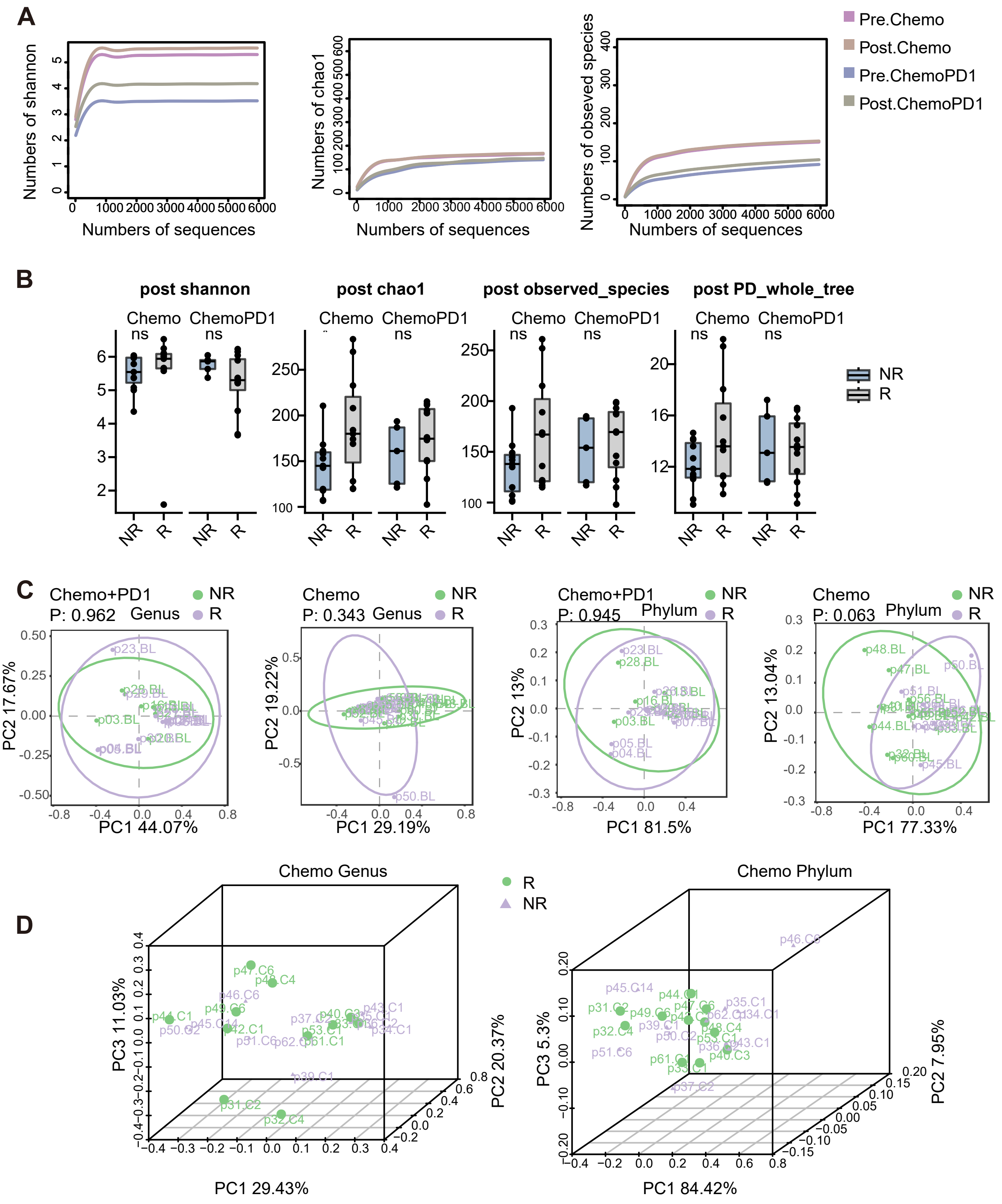

A
Pre.Chemo
Post.Chemo
Pre.ChemoPD1
Post.ChemoPD1
Numbers of shannon
Numbers of chao1
Numbers of obseved species
Numbers of sequences
B
post shannon
post chao1
post observed_species
post PD_whole_tree
Chemo
ChemoPD1
ns
NR
R
C
Chemo+PD1
Chemo
P: 0.962
P: 0.343
P: 0.945
P: 0.063
Genus
Phylum
PC1 44.07%
PC2 17.67%
PC1 29.19%
PC2 19.22%
PC1 81.5%
PC2 13%
PC1 77.33%
PC2 13.04%
D
Chemo Genus
Chemo Phylum
PC1 29.43%
PC2 20.37%
PC3 11.03%
PC1 84.42%
PC2 7.95%
PC3 5.3%

FigureS1. Blood microbial diversity and community structure before and after treatment.

(A)Species accumulation curve of all groups defined by alpha diversity index (shannon, observed species, chao1 and PD_whole_tree indices).

(B)Comparison of alpha diversity indices (shannon, observed species, chao1 and PD_whole_tree indices) across four groups.

(C)3D PCoA plots showing beta diversity at the genus (left) and phylum (right) levels using Bray-Curtis distance metrics, comparing samples after chemotherapy.

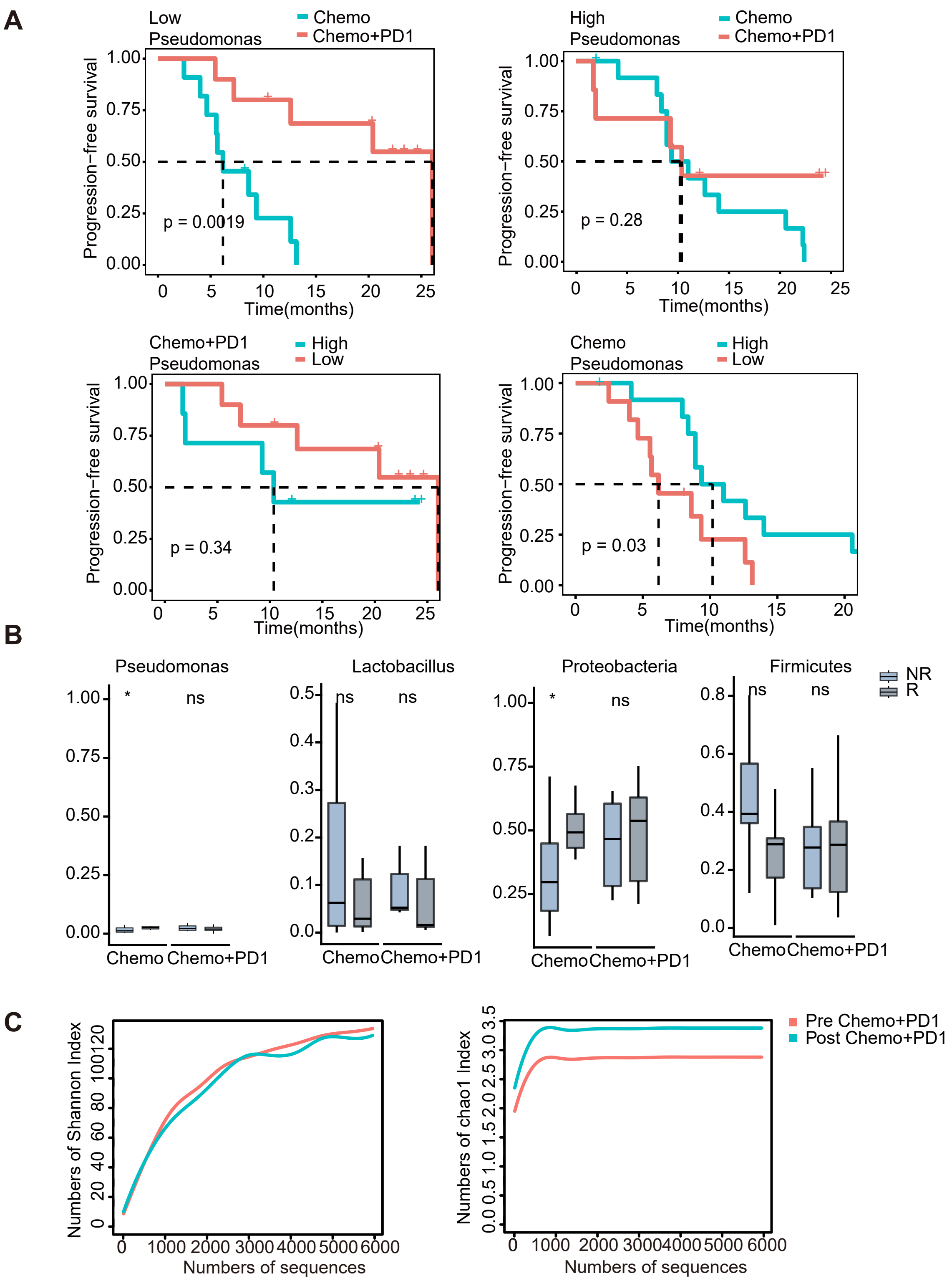
A
Low Pseudomonas
Chemo
Chemo+PD1
Progression−free survival
p = 0.0019
Time(months)
High Pseudomonas
p = 0.28
Chemo+PD1 Pseudomonas
High
Low
p = 0.34
Chemo Pseudomonas
p = 0.03
B
Pseudomonas
Lactobacillus
Proteobacteria
Firmicutes
NR
R
*
ns
Chemo
Chemo+PD1
C
Numbers of Shannon Index
Numbers of sequences
Numbers of chao1 Index
Pre Chemo+PD1
Post Chemo+PD1

FigureS2. Blood microbial abundance and survival analysis in gastrointestinal cancer patients.

(A)Kaplan-Meier survival curves of gastrointestinal cancer patients before chemotherapy (n=24) and immunotherapy combined with chemotherapy (n=17), categorized by the relative abundance of Pseudomonas.

(B)Bar chart comparing the relative abundance of Lactobacillus, Pseudomonas, Proteobacteria and Firmicutes between R and NR groups in patients before chemotherapy and immunotherapy combined with chemotherapy (Validation set 1).

(C)Species accumulation curve of all groups, defined by alpha diversity indices (shannon and chao1 indices,Validation set 1).

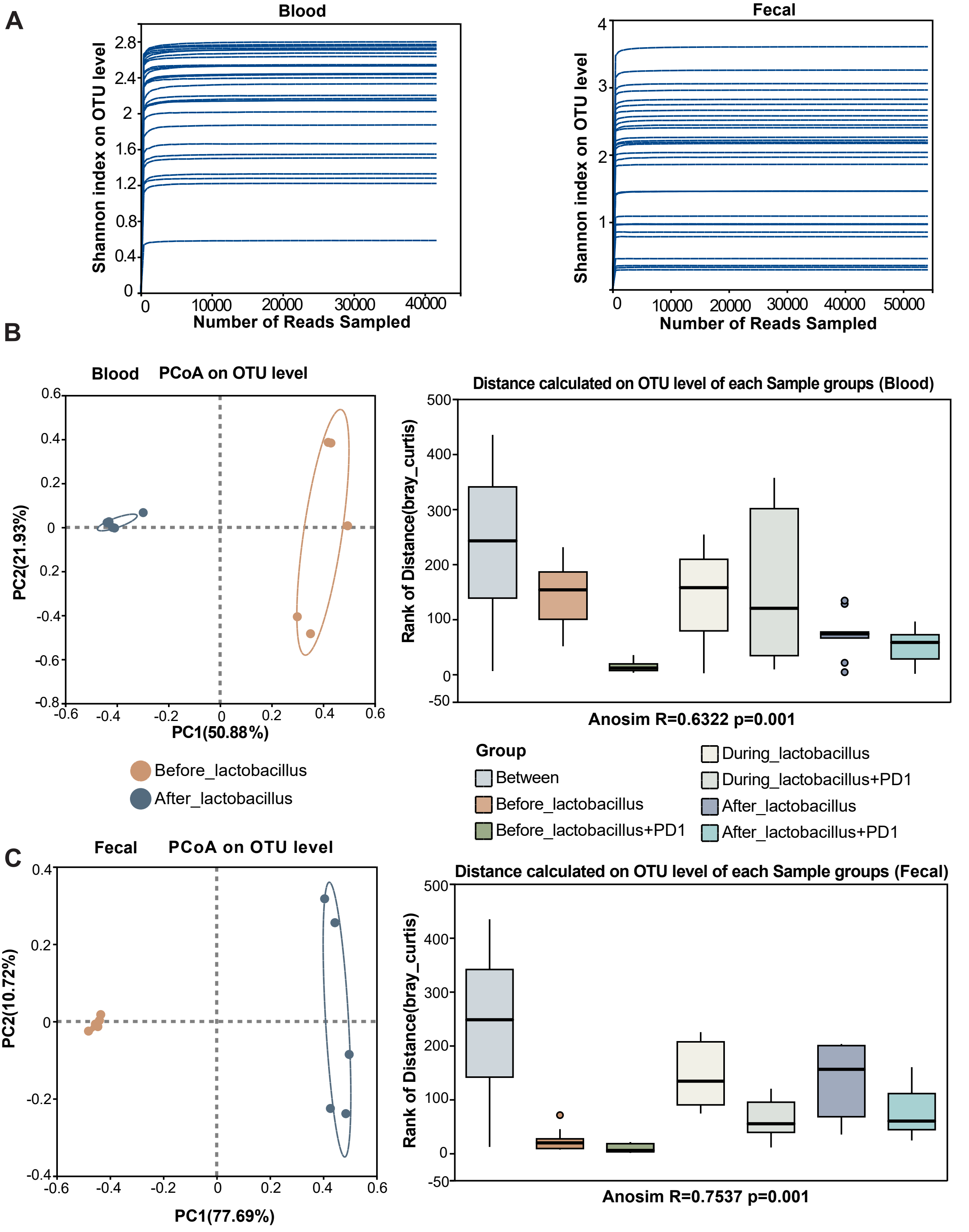
A
Blood
Shannon index on OTU level
Number of Reads Sampled
Fecal
Shannon index on OTU level
Number of Reads Sampled
B
Blood PCoA on OTU level
PC2(21.93%)
PC1(50.88%)
Before_lactobacillus
After_lactobacillus
Distance calculated on OTU level of each Sample groups (Blood)
Rank of Distance(bray_curtis)
Anosim R=0.6322 p=0.001
Group
Between
Before_lactobacillus
Before_lactobacillus+PD1
During_lactobacillus
During_lactobacillus+PD1
After_lactobacillus
After_lactobacillus+PD1
C
Fecal PCoA on OTU level
PC2(10.72%)
PC1(77.69%)
Distance calculated on OTU level of each Sample groups (Fecal)
Rank of Distance(bray_curtis)
Anosim R=0.7537 p=0.001

FigureS3. Blood microbial diversity and community structure in mouse blood and fecal samples.

(A)Species accumulation curve of all groups defined by alpha diversity (shannon index).

(B)PCoA using Bray-Curtis metric distances of beta diversity in blood sample.

(C)PCoA using Bray-Curtis metric distances of beta diversity in fecal sample.
